# Effect of glutamic acid foliar applications on lettuce under water stress

**DOI:** 10.1007/s12298-021-00984-6

**Published:** 2021-04-22

**Authors:** Giulia Franzoni, Giacomo Cocetta, Antonio Ferrante

**Affiliations:** Facoltà di Scienze Agrarie e Alimentari, Università degli Studi di Milano, Via Giovanni Celoria, 2, Milan, 20133 Italy

**Keywords:** Antioxidant, Drought stress, Foliar treatment, Glutamic acid, Re-watering, *Lactuca sativa*

## Abstract

The yield and quality of leafy vegetables can be compromised by reduced water availability. Glutamic acid is involved in different biological processes and among them it plays an important role in chlorophyll and proline biosynthesis. The aim of this work was to evaluate the possible efficacy of glutamic acid in counteracting water stress in romaine lettuce. Lettuce plants were grown in pots filled with substrate and subjected to water deprivation. A glutamic acid solution (1.9 mM) was applied as foliar treatment, both in stressed and non-stressed plants. The effect of the treatment was evaluated at different time points during the experiment in order to evaluate changes at a molecular, physiological, biochemical and agronomic level. Yield was reduced by 35% in stressed plants, while no significant changes in quality parameters were observed, except for nitrate content, which increased under water stress. At a molecular level, the expression of genes encoding for ROS scavenging enzymes was monitored but, apparently, glutamic acid did not significantly prevent the water stress response. Slightly positive effects deriving from glutamic acid application were found for nitrate and proline contents, suggesting that a possible mode of action of glutamic acid would involve a role for these molecules. Further studies are required, also on other crop species, for confirming these results. Different concentrations and application modes should be also tested.

## Introduction

Leafy vegetables are very important in the human diet, because they are a valuable source of nutrients, including fibers, minerals, carbohydrates as well as phytochemicals which are known to contribute to the health-related properties of plant derived foods (Khan et al. 2015). Therefore, growing high-quality vegetables is one of the most important goals of the current agriculture in order to meet the needs of the growing population and the increasing demand for healthy food. Among leafy vegetables, lettuce (*Lactuca sativa* L.) is one of the most popular species worldwide, cultivated either in open field or in a protected environment.

Water availability is crucial for lettuce, affecting yield and the quality of the product, especially considering that in leafy vegetables the percentage of water is very high (90–95%) (Mou 2005). Around 99% of transpired water is involved in thermoregulation, while the remaining part serves as nutrient transport and helps maintain the turgor pressure, which is associated with the textural properties of leaves (Ferrante et al. 2011). Plants can face unexpected water stress during crucial phases of the cultivation, due to environmental factors, water scarcity or non-optimal water managing. Since there is a linear relationship between yield and crop water consumption, irrigation is crucial (de Pascale et al. 2011). Thus, a more rational use of water is among the key objectives of modern cropping systems.

Plants respond to water deprivation at different levels, by showing morphological, biochemical and physiological adaptive processes. These include the stomatal closure, the synthesis of antioxidant-scavenging molecules, the activation of antioxidants enzymatic systems and the improvement of osmotic adjustment, through the accumulation of osmolytes and low weight molecules (Farooq et al. 2009; Das and Roychoudhury 2014; Rao et al. 2016; Fahad et al. 2017; Sanzón-gómez et al. 2018).

The ascorbate–glutathione pathway, also known as Halliwell-Asada cycle, is a key part of the network of reactions involving enzymes and metabolites with redox properties for the detoxification of the excess accumulation of reactive oxygen species (ROS) that occurs during stressful conditions. Ascorbate and glutathione are not consumed but take part in a cyclic transfer of reducing equivalents. The recycling process is guaranteed by the action of four enzymes (ascorbate peroxidase APX, monodehydroascorbate reductase MDHAR, dehydroascorbate reductase DHAR, glutathione reductase GR) which lead to the reduction of H_2_O_2_ to H_2_O (Noctor and Foyer 1998; Pandey et al. 2015).

Amino acids take part in plant stress responses acting as osmolytes, regulating the ion transport, the stomatal opening and they are involved in detoxification mechanisms (Rai 2002). The application of amino acids as biostimulants is a strategy that can be used in horticultural crops for counteracting the negative effects induced by environmental stresses. Amino acids can act as hormone precursors and they can contribute to regulate carbon and nitrogen metabolisms and to promote nitrogen assimilation (Miller et al. 2007; Calvo et al. 2014; Colla and Rouphael 2015; Bulgari et al. 2019). In particular, amino acids in the form of foliar spray have proved to be a promising agronomic tool (Abdelhamid et al. 2014; Teixeira et al. 2017). Studies showed that different cultivars respond to amino acids application in a different way. Moreover, the effect depend on the type of amino acids supplied as well as if they are applied in a mixture or individually (Khan et al. 2019). Besides, amino acids are utilized by plants according to their nutritional needs and environmental conditions, so the responses of plants to the same amino acid application may not always be the same.

Glutamic acid is one of the most important amino acids in plants playing a role in the biosynthesis of proline and other nitrogen-containing compounds (Okumoto et al. 2016). Amino acids are able to stimulate both primary and secondary metabolism. Several studies have pointed out the positive effect of glutamic acid application on photosynthetic activity and leaf functionality assessed through the chlorophyll fluorescence measurement (Lv et al. 2009; Serna-Rodríguez et al. 2011; Fabbrin et al. 2013; Röder et al. 2018). This is probably due to the link between photosynthetic capacity and leaf nitrogen concentration. Moreover, glutamic acid and glycine are essential metabolites playing a role in the biosynthesis of chlorophyll by being incorporated into the aminolevulinic acid (Beale et al. 1975). Cao et al. (2010) reported that exogenous application of glutamic acid improved the quality of Chinese chive and reduced the nitrate accumulation. A similar effect was observed also in lettuce plants cultivated in a hydroponic system (Haghighi 2012). Glutamic acid application had a positive effect also under stressful conditions, reducing physiological damage by enhancing the activity of antioxidant enzymes, as observed in Kimchi cabbage subjected to low temperature stress (Lee et al. 2017).

Several studies have been performed by applying a mixture of amino acids and little is known about the impact of single amino acids on plant status. The objective of the present study was to evaluate the effect of the exogenous application of glutamic acid on lettuce plants subjected to water deprivation. The hypothesis was based on the fact that the application of glutamic acid would enhance lettuce tolerance by stimulating chlorophyll and proline biosynthesis. The physiological response of plants was monitored in vivo by measuring the chlorophyll content and some chlorophyll *a* fluorescence related parameters during cultivation. Leaf nitrate, proline and osmolytes accumulation were measured as biochemical indicators of plant responses to the stress and the treatments. Moreover, the combined effect of water stress and glutamic acid was assessed at a molecular level by measuring the expression of some of the key genes encoding for the enzymes involved in ROS scavenging and ascorbate–glutathione cycle.

## Materials and methods

### Plant material, stress treatment and experimental plan

The trial was carried out at the Faculty of Agricultural and Food Science of Milan in 2018. Two-week old romaine lettuce plantlets (*Lactuca sativa* var. ‘longifolia’) were transplanted into 2.5 L plastic pots filled with a commercial substrate mixed with perlite -one plant per pot for a total of 36 plants. Plants were grown in an experimental greenhouse under controlled conditions (Temperature: 24 ± 2 °C; Relative humidity: 79 ± 12%). Nutrients were directly added to the substrate by providing 5 g of slow-release fertilizer (25:5:10 N:P:K).

**Fig. 1:**
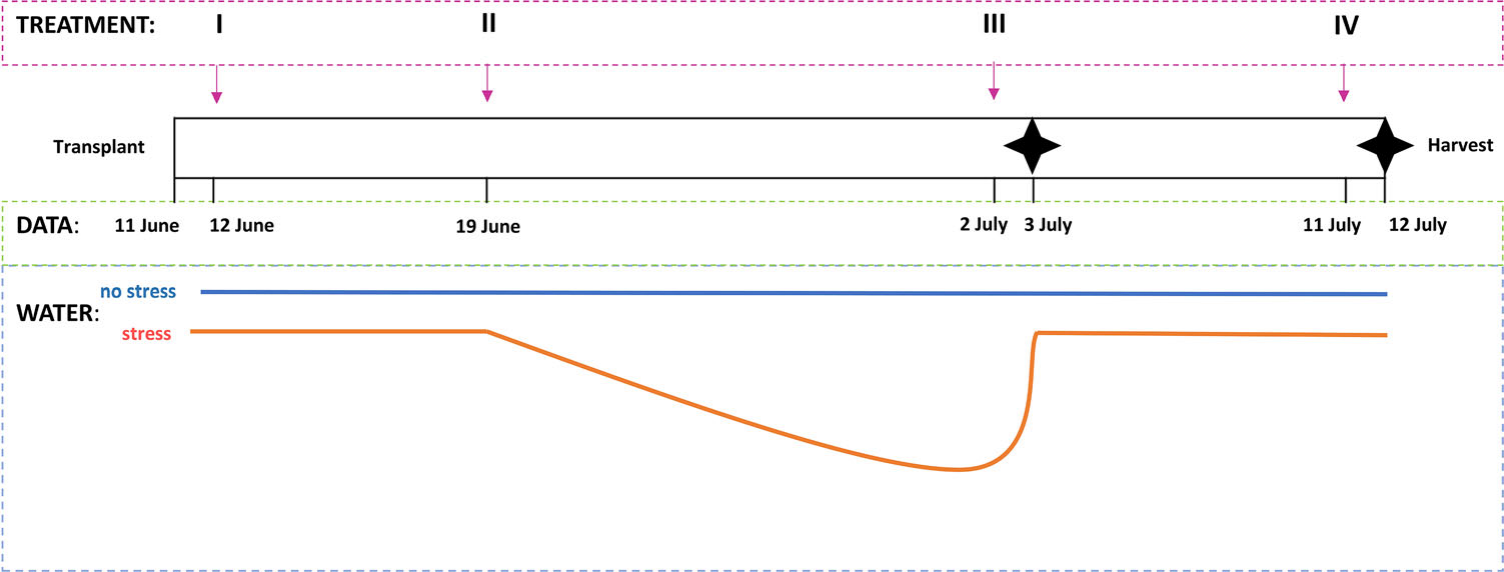
Scheme of the experimental design presenting a timeline with the timing of the treatment applications (roman numbers I, II, III, IV) and the sampling dates (indicated by the black four-pointed star symbols). The blue line indicates the well-watered plants and the red line indicates the plants subjected to a period of water deprivation followed by a re-watering period

The experimental design was based on a combination of two factors: stress (drought) and treatment (glutamic acid), each of them with two levels. Water stress was imposed 8 days after the transplant by withholding the irrigation for 15 days until the plants started to show visible symptoms of wilt and loss of turgor (Fig. S1). Soil moisture has been measured by TDR probes (WatchDog 1000 Series Micro Stations-WaterScout SM 100 Soil Moisture Sensor) and maintained constant in control plants. Moreover, the water stress response at plant level was monitored by measuring the trend of chlorophyll fluorescence parameters. In particular, the strength of the stress was evaluated as decrement of the performance index. The wilting of plants was considered as the most critical point in the experiment and the beginning of re-watering. The water supply was restored at the same level of non-stressed plants after 24 h from that moment (Fig. 1). Treatments consisted of water (control) and a glutamic acid solution (1.9 mM). The glutamic concentration applied in this experiment has been chosen based on literature review and on previous experiments (Lv et al. 2009). A Completely Randomized Design (CRD) was chosen and each experimental unit consisted of six pots. Treatments were applied as foliar spray two times before the water deprivation, 24 h before the restore of water supply, and one day before the harvest. Each plant was treated with 10 mL of product. Timesteps are reported in Fig. 1.

Samples for the gene expression analysis was collected 3 and 6 h after the third treatment and plant material was stored at −80 °C until used for RNA isolation. Sampling times for the physiological analyses are reported in Fig. 1, where they are marked with a star symbol.

### Non-destructive measurements

#### Chlorophyll in vivo

Leaves chlorophyll content was measured in vivo using a chlorophyll content meter (CL-01 Chlorophyll Content Meter, Hansatech Instruments, UK). The results are expressed as a chlorophyll index (relative units).

#### Chlorophyll *a* fluorescence

Chlorophyll *a* fluorescence was measured in vivo using two different instruments: a hand-portable fluorometer (Handy-PEA, Hansatech Instruments, UK) and a field portable pulse modulated chlorophyll fluorometer (FMS-2, Hansatech Instruments, UK). Before measurements with Handy-PEA, leaves were dark-adapted with the leaf clips for 30 min. Then were exposed to a saturating light (650 nm, 3000 μmol photons m^−2^ s^−1^) provided by an array of three high-intensity light-emitting diodes for 1 s. The measured data were used to obtain the derived parameters according to the JIP-test equations (Strasser et al. 2004). These parameters provide information about the structural and functional status of the photosynthetic apparatus and useful indication on how stress affects the distribution of energy in photosystem II (PSII). Terms, formulae and definitions of the parameters are listed in Table 1 (Strasser et al. 2004; Brestic and Zivcak 2013; Murchie and Lawson 2013; Kalaji et al. 2016, 2017).

**Table 1:** Terms and formulae used in the analysis of the fast chlorophyll *a* fluorescence

Term and formulae	Definition
Fo	Fluorescence emitted when all reaction centres (RCs) are open
Fm	Maximum fluorescence emitted when all RCs are closed
Fv = Fm—Fo	Maximum variable fluorescence
Fv/Fm = 1—(Fo/Fm)	Maximum quantum yield of primary photochemistry
Tfm	Time to reach Fm
Area	Total complementary area between fluorescence induction curve and *F* = Fm
RC/ABS	Reaction centres per adsorption of light energy
Fv/Fo	Conformation term for the primary photochemistry (curvature constant of the hyperbole)
PI	Performance index
Fo/Fm	Fluorescence of all open RCs/ Fluorescence of all closed RCs
M_0_ = TR_0_ /RC—ET_0_ /RC	Normalized value of the initial slope of the fluorescence induction curve (it expresses the net rate of the RCs’ closure)
Sm = Area/(Fm—Fo)	Normalized Area by Fv (it gives a measure of the energy needed to close all reaction centres)
Ss = (M_0_/V_J_)^−1^	Normalized Area per single turn-over
ABS/RC = (M_0_/Vj)/(Fv/Fm)	Absorption flux per RC (at *t* = 0)
TR_0_/RC = M_0_/V_j_	Trapped energy flux per RC (at *t* = 0)
ET_0_/RC = M_0_/V_j_ Ψ_0_	Electron transport flux per RC (at *t* = 0)
DI_0_/RC = (ABS/RC)—(TR_0_/RC)	Dissipated energy flux per RC (at *t* = 0)
ABS/CS ≈ Fo	Absorption flux per cross section (CS), approximated by Fo
RC/CS = (ABS/CS)/(ABS/RC)	RCs’ concentration (or density) per excited CS
TR_0_/CS = TR_0_/ABS (ABS/CS_0_)	Trapped energy flux per CS (at *t* = 0)
ET_0_/CS = ET_0_/ABS (ABS/CS_0_)	Electron transport flux per CS (at *t* = 0)
DI_0_/CS = (ABS/CS_0_)—(TR_0_/CS_0_)	Dissipated energy flux per CS (at *t* = 0)
RC/CSo	RCs’ concentration (or density) per excited CS (Fo)
RC/CSm	RCs’ concentration (or density) per excited CS (Fm)

Modulated chlorophyll *a* fluorescence under the ambient light regime was measured using the FMS-2. In order to calculate the electron transport rate (ETR) PAR value is recorded by a light sensor on the leaf-clip. The steady-state fluorescence (Fs) was measured with the measuring radiation. The effective PSII quantum efficiency (ϕ_PSII_) and the electron transport rate (ETR) were calculated by the FMS software.

### Destructive measurements

#### Yield and dry matter

Fresh weight (FW) was measured for each pot at the end of the experiment by cutting the plants at soil level and weighing the whole lettuce head. The yield was calculated considering a plant density of 10 plants per square meter. The leaf dry matter was calculated from the dry weight obtained by oven-drying samples at 105 °C until constant weight was reached.

#### Water use efficiency

The water use efficiency (WUE) was calculated as the ratio between the fresh and dry above ground biomass measured at the end of the growing cycle.

#### Nitrate

Nitrate concentration was determined by Cataldo et al. (1975) method. Fresh leaf tissue was homogenized in distilled water (1 g fresh tissue per 3 mL water). The homogenate was centrifuged at 4000 rpm for 15 min at room temperature (RT) (ALC centrifuge-model PK130R) and the recovered supernatant was used for the colorimetric analysis. Twenty microliters of the extract were added to 80 mL of 5% (w/v) salicylic acid in concentrated H_2_SO_4_ (SA- H_2_SO_4_). Afterward 3 mL of 1.5 N NaOH was added. The samples were cooled to RT and absorbance at 410 nm was measured with a spectrophotometer. Nitrate content was calculated referring to a KNO_3_ standard calibration curve. Nitrate concentration was expressed as mg of NO_3_-N per kg of FW.

#### Osmolytes

Fresh leaf tissue was homogenized in distilled water (1 g fresh tissue per 3 mL distilled water). The homogenate was centrifuged at 4000 rpm for 15 min at RT and the recovered supernatant was analysed. The osmolarity was determined using an automatic freezing point depression osmometer (Digital Osmometer, Roebling, Berlin, Germany) calibrated with sodium chloride solutions.

#### Proline

Proline concentration in leaf tissue was determined by the ninhydrin-based colorimetric assay improved by Bates, Waldren and Teare (1973). Approximately 1 g of leaf tissue was grinded with 10 mL of 3% sulfosalicylic acid. Samples were centrifuged at 4000 rpm for 5 min at RT and 100 µL of supernatant was added to a reaction mixture prepared with 3% sulfosalicylic acid, glacial acetic acid, and acidic ninhydrin. The tubes were vortexed, each lid was punctured with a needle to avoid high pressure, the tubes were incubated at 96 °C for 60 min and then the reaction was stopped by putting the tubes in ice. The extraction was made adding 1 mL toluene to the reaction mixture. The tubes were vortexed and kept on the bench for 5 min to allow the separation of the organic and water phases. The chromophore phase containing toluene was used to read the absorbance at 520 nm using toluene as reference. Proline concentration was calculated referring a standard calibration curve and it was expressed as µg per g of FW.

### Total RNA isolation and analysis of gene expression

Frozen leaves of lettuce were thoroughly ground with liquid N using a cold mortar and a pestle. Approximately 100 mg was transferred to a cryotube and stored at − 80 °C. Total RNA was isolated using the Spectrum Plant Total RNA Kit with on-column DNase-treatment (Sigma-Aldrich, Italy) following the steps of protocol A with a slight modification.

The concentration and the purity of RNA were assessed by measuring the absorbance at 230, 260 and 280 nm using a NanoDrop N-1000 spectrophotometer (NanoDrop technologies). The ratio of absorbances at 260 and 280 nm is nearly 2.0 for pure RNA and expected 260/230 values are commonly in the range of 2.0–2.2, usually higher than the respective 260/280 value.

Three μg of RNA were reversely transcribed to cDNA using the SuperScript IV cDNA Synthesis Kit according to the manufacturer’s instruction (Invitrogen, Italy).

The SYBR® Green PCR Master Mix (Applied Biosystems) was used for the quantitative RT-PCR analysis. The reaction mix was prepared by adding 10 μL of SYBR Green, 0.4 μL of forward and reverse primers, 2 μL of cDNA diluted 1:20, and 7.2 μL of RNase free water. The total volume for each PCR reaction was 20 μL. Analysis was performed using the ABI7300 (Applied Biosystem) thermocycler and PCR program and reactions were run in triplicate from two biological replicates. Gene expression analyses were performed using gene-specific primers for: superoxide dismutase [Fe] 3, chloroplastic (*LsSOD*), catalase (*LsCAT*), L-ascorbate peroxidase 6, chloroplastic/mitochondrial (*LsAPX*), monodehydroascorbate reductase, chloroplastic/mitochondrial (*LsMDHAR*), dehydroascorbate reductase (*LsDHAR*), glutathione reductase, chloroplastic (*LsGR*) (Table S1). Primers were designed using the program Primer-Blast available at the National Center for Biotechnology Information website (https://www.ncbi.nlm.nih.gov/tools/primer-blast/).

The expression levels were analysed with the AB software program and the results was calculated using the 2^−ΔΔct^ method described by Livak and Schmittgen (2001). According to this method, the data are presented as fold change in gene expression normalized to a housekeeping gene and relative to a calibrator. The Elongation factor 1 alpha (*LsEF1α*) was used as reference gene (housekeeping), whereas the non-stressed and non-treated sample after 3 h was chosen as internal calibrator.

### Statistical analyses

Data obtained from physiological analyses were subjected to a two-way ANOVA whereas data related to gene expression analysis were subjected to a three-way ANOVA. Differences among means were determined by Tuckey post-test (*P* < 0.05). Statistics were performed using GraphPad Prism for Windows (GraphPad Software, La Jolla California USA, www.graphpad.com). Additional information is reported in each figure’s legend.

## Results

### Yield, dry matter, water use efficiency

Water stress induced a significant reduction in lettuce yield. In particular, the average yield value of non-stressed plants was 1467 g m^−2^ whereas plants subjected to water deprivation had an average value of 944 g m^−2^. At the same time, the treatment with glutamic acid did not have a significant effect on yield, under both growing conditions. The same trend was observed in the dry weight and the average value of stressed plants was halved than non-stressed plants (data not shown). Similarly, the percentage of dry matter was significantly lower in stressed leaves. Water deprivation also affected the water use efficiency (WUE) and a significant decrease of about 30% was observed in plants grown under stressful condition and treated with the glutamic acid solution (Table 2).

**Table 2:** Yield, dry matter and water use efficiency (WUE) of lettuce treated with water (CONTROL) and glutamic acid and grown under two water regimes (well-watered: NO STRESS and water stress and re-watering: STRESS)

Stress	Treatment	Yield (g m^−2^)	Dry matter (%)	WUE
No stress	CONTROL	1443.3 ± 35.3 a	5.4 ± 3.1 ab	38.0 ± 0.9 ab
	GLUTAMIC ACID	1490.0 ± 12.5 a	5.8 ± 3.3 a	39.2 ± 0.3 a
Stress	CONTROL	968.9 ± 74.2 b	4.5 ± 2.6 b	32.3 ± 2.5 ab
	GLUTAMIC ACID	920.0 ± 61.3 b	4.6 ± 2.7 b	30.7 ± 2.0 b

Measures were taken at the end of the growing cycle (12/07). Values are means ± SE (*n* = 3). Data were subjected to two-way ANOVA. Different letters, where present, represent significant differences (*P * < 0.05)

### Chlorophyll and chlorophyll a fluorescence

Water deprivation induced a slight but not significant decrease in chlorophyll levels measured during the most critical point of water stress (Table 3). However, at the end of the cycle after the recovery period, chlorophyll content in stressed plants increased and reached the same values measured in plants grown with a constant water supply.

**Table 3:** Chlorophyll content determined in vivo*,* in lettuce leaves treated with water (CONTROL) and glutamic acid and grown under two water regimes (well-watered: NO STRESS and water stress and re-watering: STRESS)

Stress	Treatment	Chlorophyll (r.u.)	
		3/07	12/07
No stress	CONTROL	7.23 ± 0.63	8.38 ± 0.79
	GLUTAMIC ACID	8.70 ± 3.13	8.00 ± 0.86
Stress	CONTROL	6.26 ± 0.77	8.79 ± 0.66
	GLUTAMIC ACID	6.60 ± 0.93	8.27 ± 0.68

Measures were taken during the water stress (3/07) and at the end of the cycle after the re-watering (12/07). Values are means ± SE (*n* = 15). Data were subjected to two-way ANOVA. Different letters, where present, represent significant differences (*P*  < 0.05)

**Fig. 2:**
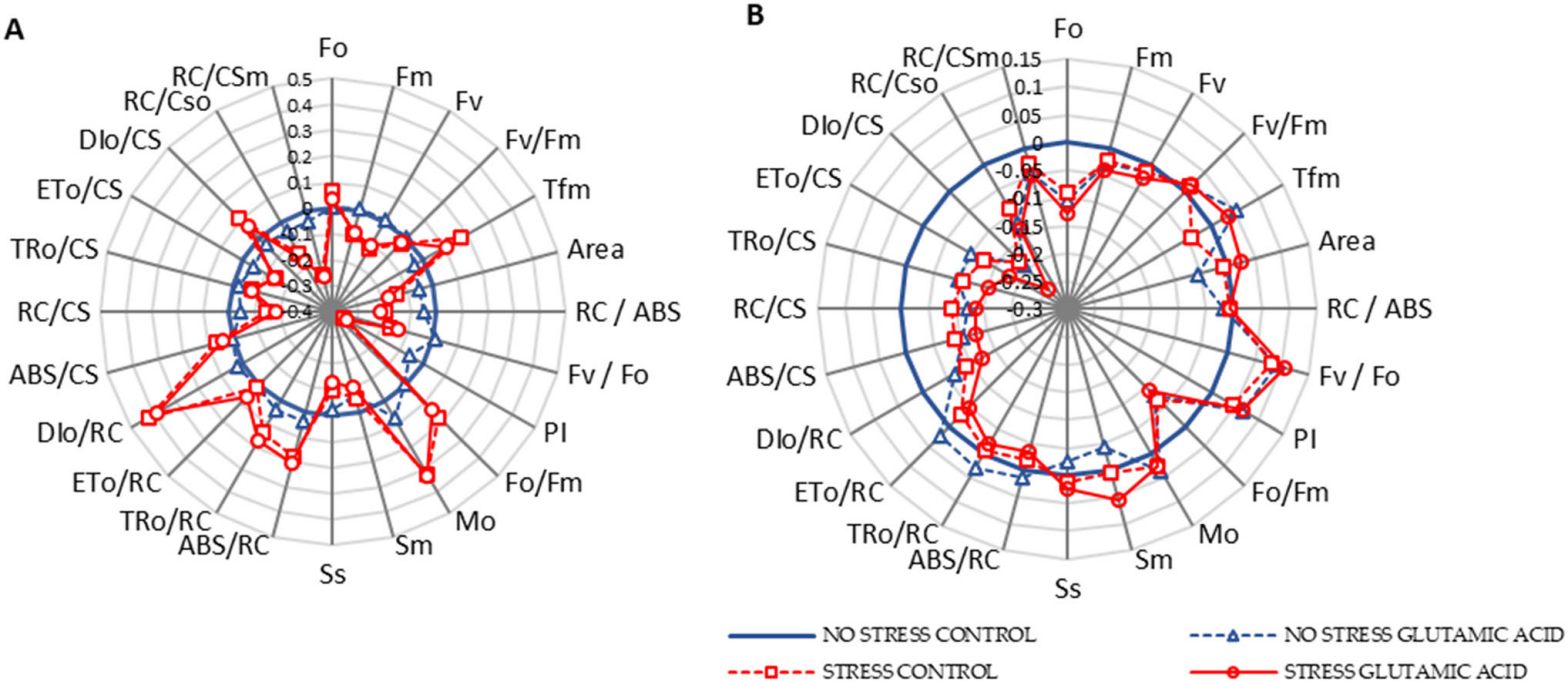
Chlorophyll a fluorescence parameters of lettuce leaves, treated with water (CONTROL) and glutamic acid and grown under two water regimes (well-watered: NO STRESS and water stress and re-watering: STRESS). Measures were taken during the water stress (3/07) **A** and at the end of the cycle after the re-watering (12/07) **B**. Data plotted are fluorescence parameters normalized by formulae: (Ft—Fnsc)/Fnsc, where “Ft” and “Fnsc” represent the parameter values of the treated plants and no stress control plants, respectively. Values of “Fnsc” plants were normalized to 0 (NO STRESS CONTROL, blue circle = 0)

Multiparametric plots of fluorescence parameters show an overall response of photosynthesis to water stress and glutamic acid treatment (Fig. 2). In these plots all the parameters are normalized to 0 -blue reference line represents the non-stressed and non-treated plants.

During water deprivation a modification of several parameters was induced, as shown in Fig. 2A. On the contrary, the application of glutamic acid did not modify any trend, regardless the water supply. Drought stress resulted in the down-regulation of PSII function, as shown by the variation of RC/CSm, DI_0_/RC, M_0_ and PI values. These parameters indicate a deactivation of reaction centres (−26%), an increase in the effective dissipation of energy per active centres (+ 40%), a high net rate of the centres’ closure (+ 33%) and a decrease in the performance index (+ 35%), respectively. The variations of the other fluorescence parameters compared with the “no stress control” were lower than 20%. The two-way ANOVA for almost all fluorescence parameters showed a significant effect of the stress condition, whereas both the interaction between the two factors and the treatment were not significant (Table S2). Similarly, the maximum quantum efficiency of PSII expressed by the Fv/Fm ratio was about 0.86 in plants growing under constant water supply, while it significantly decreased to 0.84 value in stressed plants. The time necessary to reach maximal fluorescence (Tfm) of lettuce plants was higher in stressed plants during the water deprivation if compared to well-watered control. A significant increase was observed only in control plants and not in plants treated with the glutamic acid solution. After the re-watering period fluorescence parameters stabilized and reached values more similar to those measured in non-stressed and non-treated plants, as shown in Fig. 2B. Nevertheless, the energy dissipation flux per RC (DI_0_/RC) showed a significant increase in response to water stress during the water deprivation whereas it was significantly lower after the water regime was restored. The fluorescence emitted when all reaction centres (RCs) are open (Fo), the absorption flux per cross section (ABS/CS), the ration between the fluorescence of all open RCs and the fluorescence of all closed RCs (Fo/Fm), and the dissipated energy flux per CS (DI_0_/CS) were significantly affected by the treatment at the end of the growing cycle (Table S2).

The measurement of the chlorophyll *a* fluorescence in real conditions at the end of the growing cycle showed similar results (Table 4). Indeed, both the effective PSII quantum efficiency (ϕ_PSII_) and the electron transport rate (ETR) showed that the photosynthetic apparatus functionality fully recovered after the re-watering. Moreover, a significant interaction between the stress and the treatment appeared in ETR analysis (Table S3). In contrast, a significant effect of water deprivation appeared in the analysis of steady-state fluorescence (Fs). In particular, the value measured in non-stressed plants treated with water was significantly higher than those measured in stressed plants.

**Table 4:** Effective PSII quantum efficiency (ϕ_PSII_), electron transport rate (ETR) and steady-state chlorophyll fluorescence (Fs) in lettuce leaves treated with water (CONTROL) and glutamic acid and grown under two water regimes (well-watered: NO STRESS and water stress and re-watering: STRESS)

Stress	Treatment	ϕ_PSII_	ETR	Fs
No stress	CONTROL	0.77 ± 0.01	25.9 ± 0.95	605.1 ± 21.0 a
	GLUTAMIC ACID	0.78 ± 0.01	25.2 ± 0.64	568.9 ± 11.8 ab
Stress	CONTROL	0.79 ± 0.00	23.2 ± 0.95	521.6 ± 14.7 b
	GLUTAMIC ACID	0.79 ± 0.00	26.5 ± 1.14	541.7 ± 8.9 b

Measures were taken at the end of the cycle after the re-watering (12/07). Values are means ± SE (*n* = 15) Data were subjected to two-way ANOVA. Different letters, where present, represent significant differences (*P* < 0.05)

### Nitrate, proline and osmolytes

The concentration of nitrate in lettuce leaves measured during stress was significantly affected by the water supply (Table S3). In particular, under non stressful conditions the average value was 4386 mg kg^−1^ FW whereas in stressed plants the nitrate concentration reached the value of 8559 mg kg^−1^ FW. Under stress condition the glutamic acid determined a lower nitrate concentration, even if the difference was not significant (Table 5).

**Table 5:** Nitrate content, proline and osmolytes concentrations measured in lettuce leaves treated with water (CONTROL) and glutamic acid and grown under two water regimes (well-watered: NO STRESS and water stress and re-watering: STRESS)

Stress	Treatment	Nitrate (mg kg^−1^ FW)	Proline (µg g^−1^ FW)	Osmolytes (mOsm kg^−1^ g^−1^ FW)
No stress	CONTROL	4812.7 ± 1713.7 b	20.2 ± 2.3 b	0.100 ± 0.01 b
	GLUTAMIC ACID	4213.0 ± 988.5 b	23.5 ± 1.7 b	0.098 ± 0.00 b
Stress	CONTROL	9171.5 ± 840.9 a	438.0 ± 83.6 a	0.193 ± 0.01 a
	GLUTAMIC ACID	7139.6 ± 449.9 a	455.5 ± 132.2 a	0.190 ± 0.01 a

Measures were taken during the water stress (3/07). Values are means ± SE (*n* = 3). Data were subjected to two-way ANOVA. Different letters, where present, represent significant differences (*P * < 0.05)

The concentration of proline and osmolytes in lettuce leaves during water deprivation was significantly affected by the stress (Table S3). In particular, the average level of proline in plants grown under constant water supply was about 18 µg g^−1^ whereas in those grown under water stress it strongly increased, reaching the value of 451 µg g^−1^. Similarly, the average concentration of osmolytes was 0.094 mOsm kg^−1^ g^−1^ in non-stressed plants and 0.194 mOsm kg^−1^ g^−1^ in stressed plants (Table 5).

### Gene expression

**Fig. 3:**
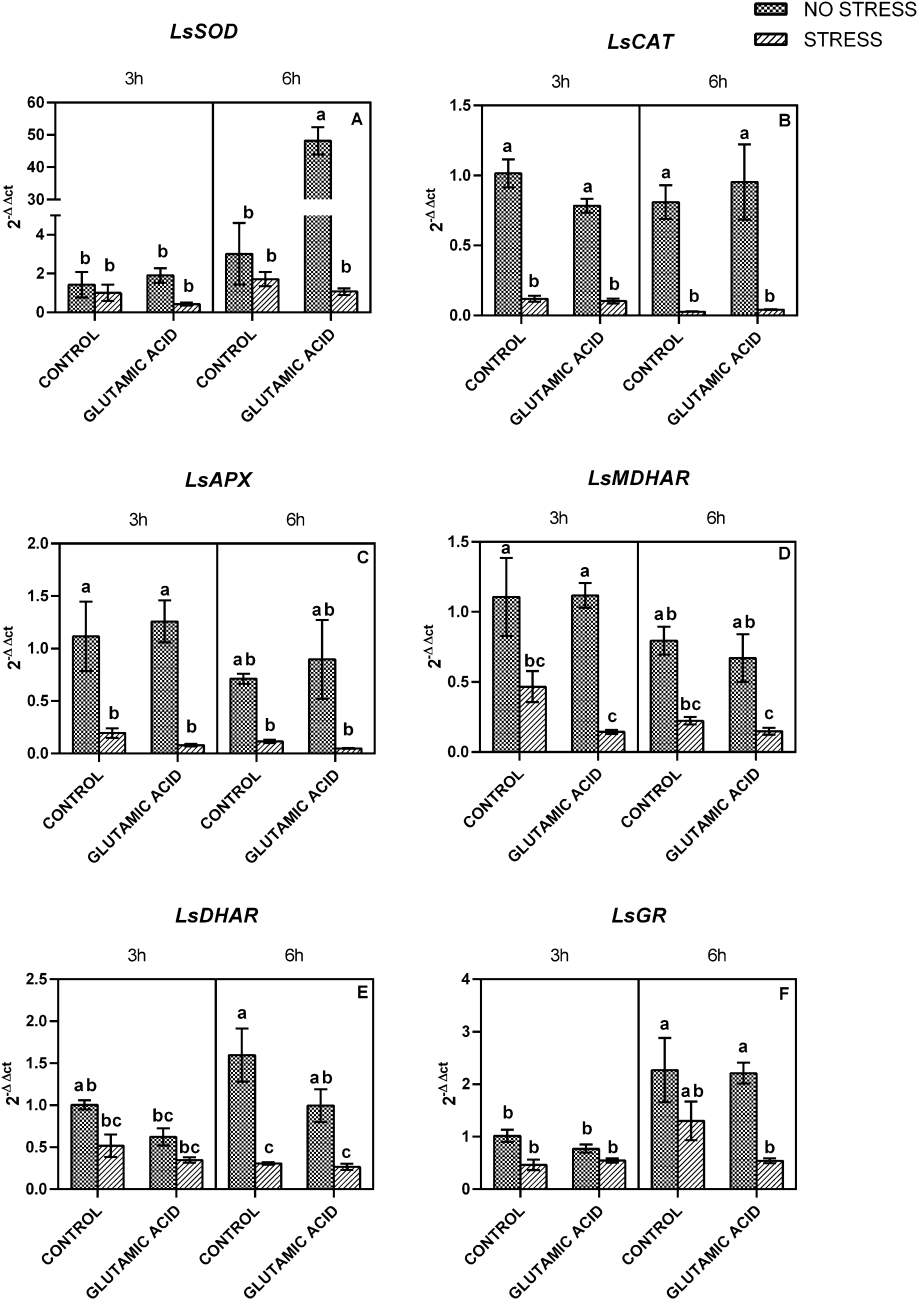
Changes in the expression of *LsSOD* **A**, *LsCAT* **B**, *LsAPX* **C**, *LsMDHAR* **D**, *LsDHAR* **E**, *LsGR* **F** in lettuce leaves treated with water (CONTROL) and glutamic acid and grown under two water regimes (well-watered: NO STRESS and water stress and re-watering: STRESS. Measures were taken 3 and 6 h after the third treatment, before the re-watering. Values are means ± SE (*n* = 6). Data were subjected to three-way ANOVA. Different letters, where present, represent significant differences (*P* < 0.05)

Water stress induced a general downregulation of the genes involved in the ascorbate glutathione cycle and ROS detoxification (Fig. 3). In particular, the expression levels of *LsCAT*, *LsAPX,* and *LsMDHAR* were significantly affected by the water deprivation whereas a significant interaction between stress and time was found for *LsDHAR* and *LsGR*. Moreover, stress*treatment interaction was significant for *LsDHAR* expression. In non-treated plants, a significant increment in *LsGR* and *LsSOD* genes expression was observed in time, with values increasing 2 and almost 3 times, respectively. On the other hand, no significant change was observed in the other genes. Stress condition induced a decrease of all genes expressions levels, both after 3 and 6 h. Treatment with glutamic acid did not induce significant changes, neither under stressful nor under optimal growing conditions, except for *LsSOD.* In particular, a peak of expression about 50 times higher was observed in non-stressed plants after 6 h from the application of the treatment.

## Discussion and conclusion

Generally, leafy vegetables need constant soil moisture levels and require a high amount of water during the entire growing cycle (Gil et al. 2012; Kirnak et al. 2016). Irrigation is essential to maximize yield, increase the turgidity of the leaves and minimize postharvest losses. In this study, we observed that water deprivation had a significantly negative impact on romaine lettuce, causing a drop of 35.7% in the yield. Plants subjected to water stress did not produce the same biomass as that produced by plants grown under optimal irrigation despite the subsequent re-watering. The same response has been observed also for the dry weight, thus its reduction accounted for the lower biomass in the stressed plants, confirming a minor accumulation of photosynthates and derived molecules. The stomata closure is a common mechanism of protection against water stress and it can help to limit water transpiration and the subsequent limitation of photosynthesis. However, this strategy leads to a reduction in the crop yield (Waśkiewicz et al. 2016). Thus, water deprivation is a limiting factor for plant growth and these results highlight the importance of a continuous water supply in leafy vegetables. These results are in agreement with those observed by Karam et al. (2002) and Sayyari et al. (2013), in lettuce plants grown under different water deficit regimes. The effect of water stress on lettuce has been extensively studied, whereas the evaluation of crop response to the combination of water shortage and re-watering is quite scarce (Karam et al. 2002; Bozkurt et al. 2009; Sayyari et al. 2013). In particular, the response of plants varies and depends on the intensity of the stress and the duration of the recovery time. For example, Oh, Carey and Rajashekar (2019) have shown that regulated water deficit positively affects phytochemical concentration in lettuce without any adverse effect on growth. The different results obtained in our experiment might be due to the severity of the stress and to the duration of the re-watering period, which in this case were longer if compared to the work described by Oh, Carey and Rajashekar, (2019). At the same time, the treatment with glutamic acid solution did not affect the yield. This might suggest that the dose of glutamic acid (1.9 mM) applied in this experiment does not alter the primary metabolism in a relevant way.

Tripolskaja and Razukas (2019) showed that the application of a mixture of glutamic acid and potassium phosphate (GAA-H_2_SO_4_) induced an increase in nitrogen and a decrease in carbohydrate concentrations in potato leaves. Moreover, the yield of mini-tubers increased in plants treated with the same mixture. Different studies showed that poly glutamic acid and other polyaminoacids promote plant growth (Xu et al. 2013, 2014, 2017; Zhang et al. 2017).

Various experiments evaluating the effect of exogenous glutamic acid applied by foliar spray have been conducted (Wang et al. 2006; Lv et al. 2009; Mazher et al. 2011; Wahba et al. 2015; Welinski de O. D’Angelo et al. 2017; Talukder et al. 2018). Lv et al. (2009) observed that several applications of a glutamic acid solution (5.44 mM) positively affected the chlorophyll content and chlorophyll *a* fluorescence parameters in hawthorn plants. This makes sense since this amino acid is a precursor in the biosynthesis of chlorophyll. In our experiment the chlorophyll *a* fluorescence as well as the level of chlorophyll were most affected by the stress rather than by the treatment. The Fv/Fm ratio is often used as stress marker and 0.83 is generally considered as the optimal value for non-stressed tissues (Maxwell and Johnson 2000). In this study the Fv/Fm of stressed plants was 0.84 whereas in non -stressed plants the average value was 0.86 during the water deprivation. This means that, even though the water reduction did not determine an impairment in the photosynthetic apparatus, its functionality and the level of chlorophyll were declining as affected by two weeks of water deprivation.

However, at the end of the growing cycle Fv/Fm values were about 0.86 in all samples and chlorophyll content reached the same level of non-stressed plants, indicating that there was no permanent damage to the photosynthetic apparatus in lettuce plants. This was further confirmed by Fo value, measured at the end of the growing cycle and by the analysis of the effective quantum efficiency of PSII (ϕ_PSII_) and by the performance index (PI). The stabilization of Fv/Fm and the decreased values of Fo are indicators of no photoinhibition and photodamage in the vegetable tissues (Yuan et al. 2013). The relation between water stress and Fs is currently exploited to have a rapid assessment of the plant status, mostly at canopy level (Flexas et al. 2000; Dobrowski et al. 2005). Unlike Fv/Fm and PI, the steady-state fluorescence (Fs) of control plants subjected to the stress was significantly lower than non-stressed plants at harvest time. A similar trend has been observed by Šajbidorová et al. (2019) and Souza et al. (2004) by evaluating the recovery of different plants after a water stress event. Stressed plants showed lower levels of ABS/CS at the end of the growing cycle. It reflects a high density of inactive reaction centres in response to drought stress, as observed in quinoa plants by Fghire et al. (2015). Moreover, the reduction in PSII activity was also confirmed by the decrease in TR_0_/CS and ET_0_/CS in stressed plants, even after the re-watering period, indicating the conversion of active RCs into inactive RCs. Due to the increase in the inactive centres, the specific fluxes per RC increased during the water stress, as shown by the high levels of DI_0_/RC, TR_0_/RC, and ABS/RC.

Glutamic acid has an essential role in amino acids metabolism and in the assimilation of ammonia in plants (Forde and Lea 2007). Moreover, Liu, Zhao and Yu (2011) reported that the main pathway for the synthesis of proline under water stress is from glutamic acid. Thus, the amount of glutamic acid provided by the treatments might have been involved in the mechanisms to cope the negative effects of the water stress, rather than the synthesis of chlorophyll, even though under stressful condition no significant difference resulted in plants treated with this amino acid. In the present experiment, the levels of proline and osmolytes were significantly higher in plants during stress. The accumulation of osmolytes such as soluble sugars, amino acids and other compatible solutes is a typical plant response to water stress. Their role is essential to protect the cellular machinery and to facilitate the osmotic adjustment (Wang et al. 2003; Iqbal and Nazar 2016; Sharma et al. 2019). In this context, proline accumulation is one of the first responses to water deficit. Anjum et al. (2011) reported that in maize plants proline level increases with the progression of drought, reaching a peak after 10 days, and then decreases when the stress becomes more severe. Furthermore, it also acts as a signaling molecule triggering the expression of specific genes (Szabados and Savouré 2009) and contributing to scavenging free radicals (Ashraf and Foolad 2007). Thus, the high level of proline in stressed plants observed in the present experiment might be an indication of the enhanced plant tolerance to water stress, induced by glutamic acid exogenous application.

The high level of nitrate measured under water stress condition might be due to a decrease activity of the nitrate reductase enzyme. Indeed, it is known that the activity of this enzyme is inhibited when soil moisture decreases, as observed in several crops. Another reason of this increase could be related to the role of nitrate as an osmotic regulator (Burns et al. 2010). The concentration of nitrate in leafy vegetables is subject to the European regulation. The threshold value for lettuce is 4000 mg kg^−1^ FW according to the harvesting periods and the growing environment. The results obtained regarding nitrate content were slightly higher in plants grown under non-stressful conditions and two times higher in stressed plants. However, the measurement was conducted during the water deprivation and not at harvest. It is known that nitrate accumulation is generally high in young leaves and we can suppose that nitrate concentration would decrease after the restore of water (Hikosaka et al. 1994).

In order to evaluate the effects of water stress on plants, their recovery performance after the re-watering and the effect of glutamic acid treatment, the expression of the genes involved in the ascorbate–glutathione cycle and in the ROS scavenging has been studied. Generally, drought stress affects photosynthetic activity and leads to photoinhibition that is associated with enhanced levels of ROS. Since ROS are toxic at high concentrations, plants react by enhancing the enzymatic and non-enzymatic antioxidant systems in order to keep ROS levels under control, and avoid oxidative damages (Gill and Tuteja 2010). Several authors reported that the expression of the genes encoding for the enzymes involved in ROS detoxification changes among plant species, and according to the stress intensity and duration (Mirzaee et al. 2013; Lum et al. 2014; Sanzón-gómez et al. 2018; Schneider et al. 2018; Rigui et al. 2019). Moreover, considering the presence of several isoforms in cytosol, mitochondria and chloroplast, a different behavior among different isoforms can be observed (Zhang and Kirkham 1996).

Lettuce leaves were sampled 15 days after the suspension of irrigation and 3 and 6 h from the third treatment with the glutamic acid solution. The expression of all genes was significantly lower in stressed plants if compared with the non-stressed ones, regardless the treatments or the sampling time. Similar results have been observed by Koffler et al. (2014) in Arabidopsis. Leaves of Arabidopsis showed the first signs of drought stress 7 days after the suspension of irrigation when turgor pressure started to drop. This phenomenon was accompanied by a general decrease in glutathione in chloroplasts, peroxisomes and the nucleus, and followed by a decrease in ascorbate. The low concentration of glutathione and ascorbate was also correlated with a suppressed activity of enzymes involved in ascorbate–glutathione cycle (GR, APX and DHAR). This led to an accumulation of ROS, plants chlorosis and necrosis. Even though in this trial the ascorbate and glutathione levels were not measured in lettuce leaves during water stress, it is possible to suppose a similar phenomenon. Due to the differences between Arabidopsis and lettuce species the wilting has been observed after different time periods of water deprivation. Moreover, in the present experiment, the stressed condition was stopped when the wilt symptoms appeared, and it has not been observed a significant decrease in chlorophyll content or leaves necrosis. A similar result has been reported by Ma et al. (2011) in apple leaves when a severe drought stress condition induced a temporary decrease in the activity of these enzymes followed by an increase after a re-watering period.

Interestingly, glutamic acid treatment induced a peak in *LsSOD* expression 6 h after its application only in plants grown under constant water supply. SOD catalyzes the dismutation of superoxide anion to hydrogen peroxide and its upregulation is usually involved in counteracting oxidative burst due to abiotic stress. Although we did not measure the enzyme activities, the abundance of *LsSOD* mRNA transcripts suggests a higher generation of superoxide in chloroplasts. This hypothesis would also indicate an overproduction of superoxide anion, which is the basic substrate for the reaction. The major site of superoxide anion production is the thylakoid membrane of photosystem I (PSI) where it is produced via the reduction of oxygen even under non-stressful conditions (Ogawa et al. 1995). However, no damage to the PSII caused by ROS emerged from the analysis of chlorophyll *a* fluorescence parameters in plants treated with glutamic acid. Moreover, according to this hypothesis, the high levels of *LsSOD* expression, along with the lower levels of *LsAPX* expression would suggest a possible accumulation of H_2_O_2_ in chloroplasts and as a result, higher damages to the cells. Nevertheless, Asada (2006) reported that H_2_O_2_ usually does not accumulate in intact chloroplasts. Glutamic acid is a common amino acid present in different organic matrix and biostimulants. Therefore, the study on its biological function could be useful for improving crop cultivation. Unfortunately, the hypothesis in lettuce has not been confirmed and the lack of significant results could be due to the species-specific responses or to the concentration of glutamic acid used (El-sharabasy et al. 2015).

Based on the results obtained in this experiment and, particularly on the gene expressions, it might be interesting to focus on SOD to clarify its role under non-stressful conditions, and its possible link to glutamic acid metabolism. Furthermore, the isoform of the genes chosen in this experiment were located in chloroplast or mitochondria, so it would be also interesting to evaluate the expression of other isoforms located in different cell compartments and at different time points, in order to understand if they increase right after the water deprivation or during the re-watering period. Finally, further experiments could be performed on other crop species by testing different modes of application, in order to better understand the mechanism of action of exogenous glutamic acid and its possible practical applications.

## Supplementary Information

Below is the link to the electronic supplementary material.

Supplementary file1 (PDF 560 KB)
