# Supplementary material for: Effect of glutamic acid foliar applications on lettuce under water stress
Source: Physiol Mol Biol Plants. 2021 Apr 22;27(5):1059–72. doi: 10.1007/s12298-021-00984-6 (PMC8140180; doi:10.1007/s12298-021-00984-6)
Supplement: Supplementary file 1 — Supplementary file1 (PDF 560 KB) [file 12298_2021_984_MOESM1_ESM.pdf]

Supplementary material

Table S1. Primers sequences and melting temperature (Tm) for qRT-PCR analysis

GENE	PRIMER PAIR	SEQUENCE (5'->3')	Tm (°C)
APX	Forward primer	ACTCATCCGGAAAGAGAGAGC	63.7
	Reverse primer	CTGAAGAGGCTTATCCGGGC	67.0
MDHAR	Forward primer	GCTTCTGTTGAGGAAGCCCT	64.7
	Reverse primer	GCACATCCTGACCTCTCTCG	65.1
GR	Forward primer	GCAATAGAGTGGAGTCAGGTGG	64.8
	Reverse primer	AACGTCTCCAACTGCCCAAA	66.7
SOD	Forward primer	ATCCATGCAACCAGGAGGTG	67.2
	Reverse primer	AAAACAAGCCAAACCCAGCC	67.1
CAT	Forward primer	AGCTTCCTGCAAATGCTCCT	65.2
	Reverse primer	GAGCAGGGTCGTGTCTTGAA	65.5
DHAR	Forward primer	CTGGATGGGCACCAAAGGTT	65.8
	Reverse primer	GACCCAATAGCATCACAAACCA	65.6
EF1α	Forward primer	TCTTGGTAGACGCCTTCACG	65.3
	Reverse primer	AGGAAGCGGTGTCATTGTTG	65.0

Table S2. Statistical significance (P) of stress (S), treatment (T), and S x T interaction on chlorophyll a fluorescence parameters.

**Parameter**	**P**					
	**3/07**			**12/07**		
	**Stress (S)**	**Treatment (T)**	**Interaction (SxT)**	**Stress (S)**	**Treatment (T)**	**Interaction (SxT)**
Fluorescence emitted when all reaction centres (RCs) are open (**Fo**)	0.0815	0.6681	0.5853	0.1200	0.0469*	0.3461
Maximum fluorescence emitted when all RCs are closed (**Fm**)	0.0103*	0.7780	0.9909	0.4354	0.3280	0.8177
Maximum variable fluorescence (**Fv**)	0.0038**	0.7217	0.9566	0.6117	0.5347	0.9773
Maximum quantum yield of primary photochemistry (**Fv/Fm**)	<0.0001 ****	0.4204	0.5634	0.1501	0.0759	0.2939
Time to reach Fm (**Tfm**)	<0.0001 ****	0.4525	0.6630	0.3716	0.0605	0.7106
Total complementary area between fluorescence induction curve and F = Fm (**Area**)	0.1230	0.5975	0.8904	0.3116	0.7531	0.2194
Reaction centres per adsorption of light energy (**RC/ABS**)	0.0416*	0.6857	0.8523	0.9561	0.7971	0.7526
Conformation term for the primary photochemistry (**Fv/Fo**)	<0.0001 ****	0.4525	0.6630	0.1011	0.0568	0.2568
Performance index (**PI**)	0.0063**	0.8170	0.7383	0.6709	0.4007	0.6464
Fluorescence of all open RCs/ Fluorescence of all closed RCs (**Fo/Fm**)	<0.0001 ****	0.4427	0.5902	0.1122	0.0480*	0.2402
Normalized value of the initial slope of the fluorescence induction curve (**M_0**)	0.0542	0.7883	0.7946	0.8483	0.5759	0.5789

Normalized Area by Fv (it gives a measure of the energy needed to close all reaction centres) (**Sm**)	0.4936	0.3652	0.8777	0.0246*	0.8623	0.0424*
Normalized Area per single turn-over (**Ss**)	0.0896	0.6130	0.9331	0.2496	0.7929	0.4881
Absorption flux per RC (at t = 0) (**ABS/RC**)	0.0498*	0.7243	0.9407	0.1906	0.9980	0.5733
Trapped energy flux per RC (at t = 0) (**TR$_0$/RC**)	0.0810	0.6533	0.9725	0.3077	0.7506	0.4611
Electron transport flux per RC (at t = 0) (**ET$_0$/RC**)	0.3078	0.3879	0.5714	0.0651	0.9399	0.4250
Dissipated energy flux per RC (at t = 0) (**DI$_0$/RC**)	0.0077**	0.9898	0.8261	0.0319*	0.1337	0.6145
Absorption flux per cross section (CS), approximated by Fo (**ABS/CS**)	0.0815	0.6681	0.5853	0.1200	0.0469*	0.3461
RCs' concentration (or density) per excited CS (**RC/CS**)	0.1534	0.6642	0.9820	0.3026	0.1104	0.4403
Trapped energy flux per CS (at t = 0) (**TR$_0$/CS**)	0.2559	0.7745	0.8461	0.0382*	0.0629	0.5031
Electron transport flux per CS (at t = 0) (**ET$_0$/CS**)	0.1331	0.7874	0.7438	0.0239*	0.0776	0.5842
Dissipated energy flux per CS (at t = 0) (**DI$_0$/CS**)	0.0509	0.4095	0.8542	0.0517	0.0353*	0.3240
RCs' concentration per excited CS (Fo) (**RC/CSo**)	0.1534	0.6642	0.9820	0.3026	0.1104	0.4403
RCs' concentration per excited CS (Fm) (**RC/CSm**)	0.0224*	0.8030	0.8911	0.6902	0.4458	0.7657

Table S3. Statistical significance (P) of stress (S), treatment (T), and S x T interaction on physiological parameters measured in lettuce plants during or at the end of the growing cycle.

**Parameter**	**P**					
	**3/07**			**12/07**		
	**Stress (S)**	**Treatment (T)**	**Interaction (SxT)**	**Stress (S)**	**Treatment (T)**	**Interaction (SxT)**
Yield (g m^{-2})	-	-	-	<0.0001 ****	0.9835	0.3821
Dry matter (%)	-	-	-	0.0023**	0.3391	0.7315
Water use efficiency (WUE)	-	-	-	0.0028**	0.9079	0.4195
Chlorophyll (r.u.)	0.0590	0.2581	0.4806	0.6511	0.5514	0.9237
Φ_{PSII}	-	-	-	0.0475*	0.6783	0.6130
ETR	-	-	-	0.4304	0.1813	0.0415*
Fs	-	-	-	0.0004 ***	0.5871	0.0619
Nitrate [mg kg-1 FW]	0.0004 ***	0.0716	0.2914	-	-	-

Proline (µg g-1)	0.0006 ***	0.8973	0.9295	-	-	-
Osmolytes (mOsm kg-1 g-1)	<0.0001 ****	0.6970	0.9606	-	-	-

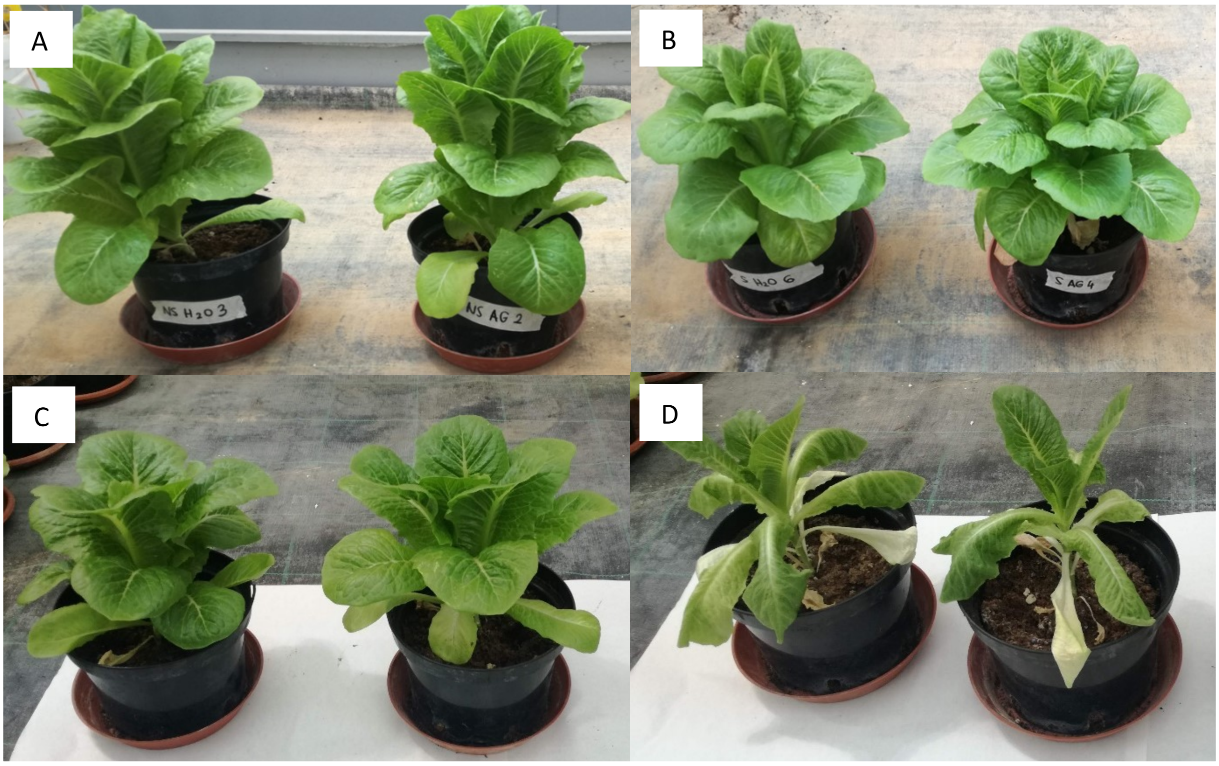

Figure S1. Lettuce plants during the water stress (3/07) (A: NO STRESS, B: STRESS) and at the end of the growing cycle after the re-watering (12/07) (C: NO STRESS, D: STRESS).
